# Use of Biosimilars to Infliximab During Pregnancy in Women With Inflammatory Bowel Disease: Results From the Pregnancy in Inflammatory Bowel Disease and Neonatal Outcomes Study

**DOI:** 10.14309/ctg.0000000000000795

**Published:** 2024-11-22

**Authors:** Millie D. Long, Sunanda Kane, Dawn Beaulieu, Bincy Abraham, Xian Zhang, Uma Mahadevan

**Affiliations:** 1 University of North Carolina, Chapel Hill, North Carolina, USA;; 2 Mayo Clinic, Rochester, Minnesota, USA;; 3 Vanderbilt University, Nashville, Tennessee, USA;; 4 Houston Methodist, Houston, Texas, USA;; 5 University of California, San Francisco, California, USA.

**Keywords:** inflammatory bowel disease, Crohn's disease, ulcerative colitis, pregnancy, biosimilar

## Abstract

**INTRODUCTION:**

We aimed to compare pregnancy outcomes of women with inflammatory bowel disease using biosimilar vs originator infliximab (IFX).

**METHODS:**

In a prospective cohort of pregnant women with inflammatory bowel disease, we collected characteristics, medications, pregnancy outcomes, and developmental milestones. We compared outcomes by IFX biosimilar or originator use via bivariate statistics.

**RESULTS:**

A total of 100 pregnant women on originator IFX and 20 on biosimilar IFX were included. There were no differences in pregnancy complications between groups (48% vs 35%, *P* = 0.29). Infant developmental milestones were comparable at 12 months.

**DISCUSSION:**

Biosimilar IFX is not associated with adverse pregnancy or infant outcomes.

## INTRODUCTION

Inflammatory bowel diseases (IBDs), including both Crohn disease (CD) and ulcerative colitis (UC), are autoimmune disorders with peak diagnosis during the reproductive years. Therefore, it is important to understand best practices for management of IBD during pregnancy to improve outcomes for both mothers and infants. Recent data have demonstrated the importance of continuation of biologic therapy during pregnancy (1,2), control of disease activity, (2) and avoidance of corticosteroids (3) to improve outcomes for women with IBD and their offspring.

Large prospective cohorts have demonstrated the safety and efficacy of antitumor necrosis factor alpha (anti-TNF) therapies during pregnancy among women with IBD (2). Infliximab (IFX) was initially approved in the United States for CD in 1998 (4) and has now demonstrated a track record of safety during pregnancy. IFX is an immunoglobulin that is actively transported across the placenta by Fc receptors during the second and third trimester. Levels can persist in infants up to 9 months after birth (5,6). Exposure to anti-TNF agents in utero has not been associated with increased serious infections (2), differing vaccine response (7), or complications in infants. The groundbreaking studies demonstrating efficacy and safety of anti-TNF agents in the United States included pregnant women with IBD on originator biologics (2).

The first biosimilar to IFX was approved in 2016 in the United States and became commercially available in 2017. The Food and Drug Administration requires that a biological product be highly similar to the reference product with only minor differences in clinically inactive compounds (4). Biological products are large (high molecular weight) compounds with complex, heterogeneous structures. Biologics are extremely dependent on the process used to create them in biological systems, and even small changes in the manufacturing process can alter their structure and potentially affect safety. Unlike chemical drugs and their derived generic compounds, biosimilars have a potential for variation from the reference product (8). These minor differences may not be clinically relevant from an efficacy perspective, but do have considerations for pregnancy. In particular, women with IBD are concerned about the safety of specific therapies during pregnancy (9). For many years after approval of biosimilars in the United States, insurance plans offered an exception for pregnancy, where pregnant women with IBD could continue on originator IFX due to limited data on the biosimilar agents and pregnancy outcomes.

We therefore sought to compare pregnancy complications, neonatal outcomes, and developmental milestones for children at 1 year in a population of women with IBD exposed to originator IFX as compared with biosimilar IFX from 2017 to 2024. We aimed to inform policy and provide data for shared decision making on biosimilar use of IFX during pregnancy.

## METHODS

The Pregnancy in IBD and Neonatal Outcomes study is an ongoing prospective observational cohort study that has enrolled pregnant women with IBD at 30 US centers since January 2007 (NCT00904878). The details of the study design have been described elsewhere (2). In brief, women with IBD and singleton pregnancies are eligible for inclusion. Questionnaires were administered at study intake, each subsequent trimester, delivery, and 4, 9, and 12 months after birth. Owing to the timeline of approval of biosimilar IFX, for this study, the cohort was limited to those individuals enrolled from 2017 to 2024 who reported use of either originator IFX or biosimilar IFX in the 3 months before last menstrual period or at any point during pregnancy.

### Exposures

Demographic data, IBD subtype, medication utilization, and disease activity were collected. Disease activity was measured using Harvey-Bradshaw Index (10) for CD and Simple Clinical Colitis Activity Index for UC (11).

### Outcomes

Pregnancy outcomes and complications of labor were collected including spontaneous abortion, preterm birth (< 37 weeks), stillbirth, intrauterine growth restriction, small for gestational age, low birth weight (< 2,500 gm), and cesarean section. Infant developmental milestones were assessed through the nationally validated *Ages and Stages Questionnaire (ASQ), Third Edition* (12), at age 12 months.

### Statistical analysis

We examined univariate and bivariate distributions of all exposures and outcomes. We compared pregnancy and neonatal outcomes among women exposed with originator IFX as compared with biosimilar IFX using bivariate statistics. For developmental milestones, we compared mean scores on each milestone by IFX exposure category through *t*-test. Data were analyzed using SAS 9.2 (Cary, NC).

## RESULTS

A total of 120 pregnant women were included with 120 birth outcomes. Of these, 100 were exposed to originator IFX and 20 were exposed to biosimilar IFX. Biosimilars used included IFX-dyyb (13/20, 65%) and IFX-axxq (7/20, 35%). Characteristics of the population were generally similar by exposure group. Overall, approximately two-thirds of the cohort had CD. The group on biosimilar IFX had a significantly longer median duration of disease when compared with originator IFX (17.0 years, interquartile range 7.0–21.0 vs 10.0 years, IQR 6.0–15.0, *P* = 0.002). Clinical characteristics by exposure group are shown in Table 1.

**Table 1.** Characteristics of the population of pregnant women in PIANO exposed to originator and biosimilar IFX 2017–2024

Characteristic	Overall		Originator IFX		Biosimilar IFX		*P* value^a^
	N = 120	% or mean (SD), median IQR	N = 100	% or mean (SD), median IQR	N = 20	% or mean (SD), median IQR	
Age (mean, SD)	120	32.3 (4.6)	100	32.0 (4.5)	20	33.8 (4.9)	0.12
Total pregnancies	120	2.1 (1.1)	100	2.1 (1.1)	20	2.0 (1.4)	0.96
Disease duration (yr)	120	10, 6.0–15.0	100	9.0, 6.0–13.5	20	17.0, 7.0–21.0	0.002
Disease type							
Crohns disease	82	68%	69	69%	13	65%	0.52
UC/IBD-U	37	32%	30	31%	7	35%	
Thiopurine use (% yes)	21	18%	17	17%	4	20%	0.75
Steroid use (% yes)	27	23%	22	22%	5	25%	0.77
Smoking status							
Never	100	83%	84	84%	16	80%	0.66
Former	18	15%	14	14%	4	20%	
Current	2	2%	2	2%	0	0	

IBD, inflammatory bowel disease; IFX, infliximab; PIANO, Pregnancy in IBD and Neonatal Outcomes; UC, ulcerative colitis.

a By χ^2^, Fisher exact, *t*-test, or Wilcoxon rank sum as appropriate.

Among women on biosimilar IFX, 63% continued on therapy through delivery, with higher continuation rates among women on originator IFX (87%). No women on biosimilar IFX had active disease during pregnancy; 5 (5%) had active disease on originator IFX.

Pregnancy-related outcomes are shown in Table 2. Overall, there were no differences in any pregnancy complication by IFX exposure status. Adverse outcomes were generally rare (all ≤ 10% except for c-section). Rates of c-section were 35% for those on IFX originator and 25% for those on IFX biosimilar (*P* = 0.39).

**Table 2.** Pregnancy-related complications by originator vs biosimilar infliximab drug exposure in PIANO cohort 2017–2024

	Infliximab (originator) (n = 100)		Infliximab (biosimilar) (n = 20		
Event					*P* value
Complication	N	%	N	%	
Spontaneous abortion gestation <= 140 d	2	2%	0	0	0.52
Spontaneous abortion any gestation	2	2%	0	0	0.52
Preterm birth (<37 wk)	5	5%	1	5%	1.0
Small for gestational age	4	4%	1	5%	0.84
Low birth weight (<2,500 g)	9	9%	1	5%	0.56
Intrauterine growth restriction	2	2%	1	5%	0.43
Caesarean section	35	35%	5	25%	0.39
Neonatal ICU	5	5%	1	5%	1.0
Any congenital malformation	7	7%	2	10%	0.64
Any of the above except c section	48	48%	7	35%	0.29

ICU, intensive care unit; PIANO, Pregnancy in IBD and Neonatal Outcomes.

Infant developmental milestones were comparable with the general population, and no difference in any milestone was seen by originator vs. biosimilar status (Table 3).

**Table 3.** Ages and stages developmental milestones by originator vs biosimilar infliximab drug exposure in PIANO cohort 2017–2024 at 1 year

12 mo milestone	Originator IFX			Biosimilar IFX			*P*-value
	n	Mean	SD	N	Mean	SD	
Communication	49	51.0	11.90	3	55.0	8.66	0.57
Fine motor	49	55.2	6.57	3	56.7	5.77	0.70
Gross motor	49	50.7	13.77	3	50.0	10.00	0.93
Personal social	49	52.8	9.27	3	53.3	5.77	0.92
Problem solving	49	50.4	10.95	3	53.3	7.64	0.65

IFX, infliximab.

## DISCUSSION

Biological agents, including anti-TNF agents, have enhanced the care of patients with IBD. These agents are associated with robust clinical remission rates and are the mainstay of therapy for moderate to severe CD or UC. However, these agents have significant costs. As biologics reached the end of market exclusivity, biosimilars have been developed. These are not exact copies, but rather highly similar to the originator biologic. Acceptable ranges of variations have been clearly defined by regulatory agencies. A biosimilar is required to be highly similar, without functional consequences for efficacy, safety, pharmacokinetic parameters, or immunogenicity (13). Biosimilar anti-TNF agents are considered appropriate for moderate to severe biologic-naïve IBD patients and for those previously treated with originator agents, to reduce costs (14).

Thus far, there have only been small case series in mixed autoimmune populations, predominantly rheumatologic diseases, of biosimilar use during pregnancy (15,16). A prior retrospective review of 18 pregnancies with exposure to biosimilar anti-TNF (including IFX, etanercept and adalimumab) at the time of conception described outcomes. In this population, only 6 women had CD and 1 with UC. Only 7 women continued their biosimilar throughout pregnancy. There were 18 lives births in this group, with 1 preterm birth (15). A separate series of 5 pregnancies in 3 women with rheumatologic diagnoses described pregnancy outcomes on biosimilars to anti-TNF (IFX, etanercept) (16). These series are small, retrospective, and without a comparator group.

Our study collected prospective maternal and infant outcomes in women with IBD on biosimilar IFX and compared these outcomes over the same period with women on originator IFX. There was no difference in any specific pregnancy complication or overall rates of complications excluding c-section (48% IFX originator vs. 35% IFX biosimilar, *P* = 0.29). Children followed over 1 year had similar developmental milestones. There were no differences in active IBD during pregnancy, with low rates in both groups.

There are a number of strengths to this study, including the prospective data collection, the objectively confirmed diagnosis of IBD, the use of validated instruments when applicable (such as for disease activity), and inclusion of pregnant women from geographically diverse areas across the United States. There are also a number of limitations. While this represents the largest series to date of pregnant women with IBD on biosimilar IFX, the sample size is still limited. In addition, owing to the timing of delivery, not all children have developmental milestones available at 1 year. While we have access to the type of biosimilar IFX for most of the included patients (IFX-dyyb), the specific type of IFX biosimilar is unknown for some participants.

Prior data from Pregnancy in IBD and Neonatal Outcomes have demonstrated the safety and efficacy of anti-TNF agents during pregnancy (2). The additional data presented here on biosimilar IFX are of utmost importance when counseling patients contemplating pregnancy where both provider and patient may have hesitation. Biosimilar IFX has now been specifically evaluated in pregnancy, with comparable outcomes with originator IFX in a population of women with IBD.

## CONFLICTS OF INTEREST

**Guarantor of the article: **Millie D. Long, MD, MPH, FACG.

**Specific author contributions:**M.D.L.: conceptualization, methodology, investigation, formal analysis, and writing—original draft. S.K., D.B., B.A.: conceptualization, recruitment of patients, data interpretation, review and approval of content. X.Z.: formal analysis, data curation, and writing—original draft. U.M.: conceptualization, methodology, data interpretation, writing—review and editing. All authors had access to the study data and reviewed and approved the final manuscript.

**Financial support:** PIANO is supported by a grant from the Leona M. and Harry B. Helmsley Charitable Trust.

**Potential competing interests:** M.D.L.: Consulting AbbVie, Takeda, Pfizer, Janssen, BMS, Lilly, Prometheus, Intercept, Target RWE, Research support: Pfizer, Lilly, Takeda. S.K.: Consultant Boerhinger Ingelheim, Bristol Meyers Squibb, Fresenius Kabi, Janssen, Lilly, Takeda. Editor for UptoDate. D.B.: Consulting: Abbvie, Bristol Myers Squibb, Celltrion, Institute of Functional Medicine, Takeda. B.A.:Consulting: Abbvie, Bristol Myers Squibb, Celltrion, Pfizer, Lilly, Takeda, Prometheus, Janssen. Speakers Bureau: Abbview, Bristol Myers Squibb, Pfizer, Lilly, Takeda, Janssen. X.Z.: None. U.M.: Consulting: Abbvie, Bristol Myers Squibb, Boeringher Ingelheim, Celltrion, Enveda, Gilead, Janssen, Lilly, Merck, Pfizer, Protagonist, Rani Therapeutics, Roivant, Takeda.
